# Progress in Orthopædics

**Published:** 1901-01-05

**Authors:** 


### PROGRESS IN ORTHOPÆDICS.

**Hip Disease.**—Shaffer[9] writes on the neuro-muscular elements in hip disease, and warns against the too prolonged use of immobility as treatment for this disease. The protective spasm of the muscles dies away and a limb previously immobile becomes slightly movable. This amount of movement he holds to be harmless. If the limb is now rendered immobile again, harm is caused. But he speaks against the removal of all apparatus before the time. He says: "I believe that in all but the exceptional cases a relapse as to the deformity or the disease, or both, is likely to occur as the result of the traumatism of ordinary locomotion, unless the proper mechanical protection is maintained until the articulation is free from true reflex muscular spasms or is ankylosed."

The writer states that the condition of tetanoid spasm round a joint proves the continuance of disease and irritation.

Barker[10] contrasts his record of 41 cases treated by operation for destructive hip-joint disease with similar notes by Croft published in 1879. In Croft's series 13.5 per cent. died as result of operation, in Barker's list there is one possible case. The after-results are good; only 3 cases, that could be traced over twenty-two years, have died from any cause. Recurrence was noted in 10 cases after some years, two after many months. Beyond shortening, which will occur without operation, the patients walked well and without pain.

Arbuthnot Lane[11] holds that traumatism is very generally in evidence as a starting-point of tubercle of the hip. Synovitis may be set up by bruising the articular surface; the layer of bone beneath the hyaline covering may be injured and inflamed; or the epiphyseal line may be the seat of injury and become infected.

Injury may occur to the growing line varying from slight bruising to a complete separation of epiphysis when the hip-joint is flexed, or flexed and adducted, or flexed and abducted.

He believes that complete separation of the epiphysis is by no means uncommon as a cause of tubercular infection —seeing that it so often occurs without causing painful symptoms. It is highly probable, however, that damage to the line, short of complete separation, is very much more frequent.

The articular cartilaginous covering of the subjacent bone and the epiphyseal line are also liable to damage when the child falls on the side, the outer surface of the trochanter striking the ground, or through the length of the femur when the child alights on the feet.

For treatment he uses hygienic measures, such as increased respiration in good air, good food, &c.; germicides, *e.g.* mercury, iodides of ammonium, potassium and sodium, and gradually increasing doses of liq. arsenicalis; as complete rest as possible to the affected part. Where there is flexion associated with adduction or abduction, double extension is advisable to render the limbs as parallel as possible. But where there is displacement of the head of the bone extension is useless, and operation must be undertaken. In extending the limb care should be taken "to ensure the normal outward rotation of the limb," exaggerating this angle of rotation to meet the tendency to backward displacement of the head of the femur with destruction of the acetabular margin.

The objects in operative treatment are: (1) complete removal of tubercular products; (2) mechanical improvement; (3) the combination of these.

He uses a transverse incision backwards from the anterior inferior spinous process of the ilium. The advantages are that the parts can be thoroughly exposed, dorsal displacement and diseased foci in femur and ilium can be reached, and a new joint can be readily made if required.

Nichols and Bradford[12] describe anatomically five specimens of congenital dislocation of hip-joint with a view of showing the obstacles to reduction, and some reasons why, when reduced without operation, the deformity tends to relapse.

The anatomical points in these cases are: (1) That the capsular ligament presents various shortened bands, chiefly laterally and below; (2) constriction of the middle of the capsule so that the head of the bone cannot be pushed through; (3) thickening of the capsule just above the apex of the acetabulum; (4) adhesions between capsule and head of femur; (5) contraction of adductor muscles; (6) an increase of femoral torsion so that when the head of the femur is placed in the normal acetabulum the knee looks inward.

Obstacles to retention of the head of femur after complete reduction are: (1) A flattened or insufficient acetabulum, placed too obliquely; (2) an insufficient head; (3) an extremely short neck of the femur; (4) obliteration of the cavity of the acetabulum by a dense mass of fibrous tissue.

The obliquity of the neck of the femur is one of the causes of relapse; this may be obviated by osteotomy below the lesser trochanter. The head must be placed well in the acetabulum (and there should be a projecting lip on the upper margin of the acetabulum). Time must be allowed for the soft parts to adjust themselves, while the limb is fixed in its new position.

**Infantile Paralysis.**—Muirhead Little,[13] treating of infantile paralysis, says from his statistics that girls are slightly more liable to the affection than boys (60:55), while the paralysis is more severe in boys. The left side is more often affected than the right (as 78:68). With the exception of two cases, said to be congenital, the age of incidence was six weeks to eleven years. Nearly one-third of the cases occurred in the second year, and in 78 cases before the end of the third year.

In 41 cases there was gradual, ill-defined onset; in 43 paralysis came on suddenly; in 19 of these cases the child was healthy when put to bed, and was found paralysed in the morning.

Teething was very rarely suggested as a cause by the parents, convulsions occurred in 4 cases, pain in 1 case, and fever in 2 only. Injury seems surprisingly negative. Influenza, vaccination, diphtheria, and sitting on damp grass were given as the cause each in one case.

In treating the cases, subcutaneous section of contracted tendons and fasciæ, correcting splints, exercise massage, and manipulations with subsequent retentive apparatus, were generally used. In only 12 feet was an open operation considered necessary, tenoplasty was performed 8 times, and arthrodesis 5 times.

**Hammer Toe.**—Warrington Haward[14] lays stress upon the bursa frequently associated with hammer toe occurring beneath the corn, the inflammation of which is the chief agent in the production of the pain of that deformity. The relief of the pressure in the bursa should be produced before operating by the insertion of a lancet point beneath the corn. After subsidence of the inflammation the corn should be excised together with the head of the proximal phalanx.

He also recommends for the treatment of hallux valgus that the metatarso-phalangeal joint should be excised with division of the extensor tendon, and the bursa should be removed. Fibrous ankylosis takes place with satisfactory results. In these cases the cartilage which has ceased to enter into the action of the joint becomes atrophied.

[9] New York Med. Jour., April 14 and 28. [10] Lancet, May 26. [11] Clin. Jour., June 20. [12] Amer. Jour. Med. Sci., June. [13] Brit. Med. Jour., Sept. 1. [14] Lancet, July 28.

